# Treatment of multilevel degenerative lumbar spinal stenosis with spondylolisthesis using a combination of microendoscopic discectomy and minimally invasive transforaminal lumbar interbody fusion

**DOI:** 10.3892/etm.2012.812

**Published:** 2012-11-14

**Authors:** HAN WU, WEI-DONG YU, RUI JIANG, ZHONG-LI GAO

**Affiliations:** 1 Departments of Orthopaedics, China-Japan Union Hospital of Jilin University, Changchun, Jilin 130033, P.R. China; 2 Neurosurgery, China-Japan Union Hospital of Jilin University, Changchun, Jilin 130033, P.R. China

**Keywords:** microendoscopic discectomy, minimally invasive transforaminal lumbar interbody fusion, posterior lumbar interbody fusion, lumbar spinal stenosis, lumbar spondylolisthesis

## Abstract

Degenerative lumbar spinal stenosis (DLSS) has become increasingly common and is characterized by multilevel disc herniation and lumbar spondylolisthesis, which are difficult to treat. The current study aimed to evaluate the short-term clinical outcomes and value of the combined use of microendoscopic discectomy (MED) and minimally invasive transforaminal lumbar interbody fusion (MI-TLIF) for the treatment of multilevel DLSS with spondylolisthesis, and to compare the combination with traditional posterior lumbar interbody fusion (PLIF). A total of 26 patients with multilevel DLSS and spondylolisthesis underwent combined MED and MI-TLIF surgery using a single cage and pedicle rod-screw system. These cases were compared with 27 patients who underwent traditional PLIF surgery during the same period. Data concerning incision length, surgery time, blood loss, time of bed rest and Oswestry Disability Index (ODI) score prior to and following surgery were analyzed statistically. Statistical significance was reached in terms of incision length, blood loss and the time of bed rest following surgery (P<0.05), but there was no significant difference between the surgery time and ODI scores of the two groups. The combined use of MED and MI-TLIF has the advantages of reduced blood loss, less damage to the paraspinal soft tissue, shorter length of incision, shorter bed rest time, improved outcomes and shorter recovery times and has similar short-term clinical outcomes to traditional PLIF.

## Introduction

With aging of the population, degenerative lumbar spinal stenosis (DLSS) is becoming an increasingly common spinal disease. DLSS is often characterized by radiological findings of multilevel disc herniation and lumbar spondylolisthesis and is difficult to treat. Although there has been a series of improvements in surgical technique, the traditional laminectomy with interbody or posterolateral fusion from the posterior approach continues to be widely used. However, multilevel fusion causes great damage to the normal structure, prolongs recovery time and may result in chronic lower back pain (1). In the current study, we evaluated the combined use of microendoscopic discectomy (MED) and minimally invasive transforaminal lumbar interbody fusion (MI-TLIF) for the treatment of multilevel DLSS with spondylolisthesis, which had satisfactory short-term clinical outcomes, and compared the combined surgery with the traditional lumbar interbody fusion from the posterior midline approach (PLIF).

## Materials and methods

### Patients

A total of 26 patients with multilevel DLSS and spondylolisthesis (the minimally invasive group) who underwent combined MED and TLIF surgery using a single-cage and pedicle rod-screw system between July 2009 and March 2011 were involved in the study. In this group, 14 patients were male and 12 were female with a mean age of 63.4 (range 53–78) years; 14 patients had stenosis of 2 segments, 12 patients had stenosis of 3 segments and all had spondylolisthesis of 1 segment. The above cases were compared with 27 patients (the traditional group) who underwent traditional PLIF surgery during the same period. In the traditional group, 15 patients were male and 12 were female; their ages ranged from 55 to 75 years with an average of 64.9 years; 15 patients had stenosis of 2 segments, 12 patients had stenosis of 3 segments and all had spondylolisthesis of 1 segment. Data regarding the incision length, surgery time, blood loss, time of bed rest and Oswestry Disability Index (ODI) scores prior to and following surgery were analyzed statistically. The study was approved by the ethics committee of China-Japan Union Hospital of Jilin University (Changchun, China).

All patients had a history of recurrent and progressive lower back pain and leg pain with intermittent claudication for 1–20 years, with an average duration of 4.5 years. They had all undergone systematic conservative treatment for at least 3 months but had experienced no improvement.

**Figure 1.**
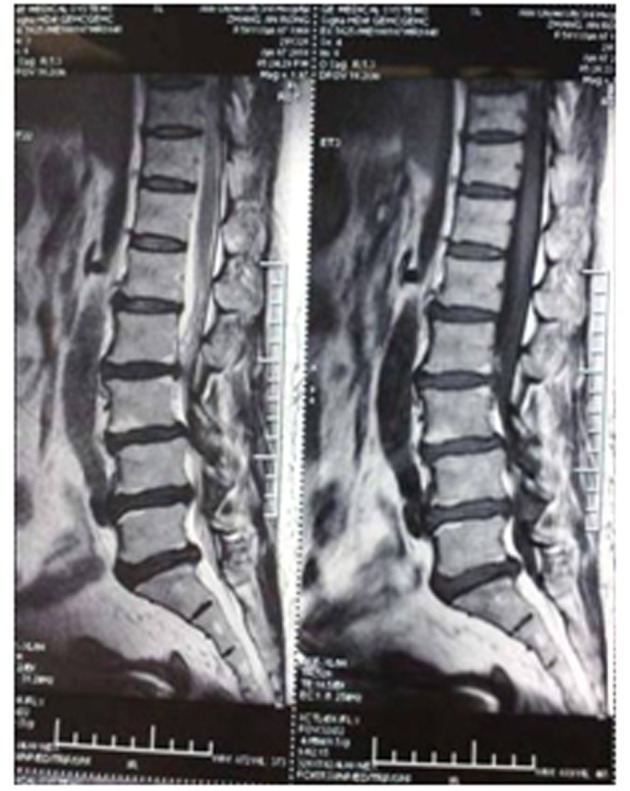
MRI revealed L3 spondylolisthesis, L45 and L5S1 disc herniation and spinal stenosis.

All patients were examined by X-ray imaging of the lumbar anterior and posterior (AP) view, lateral view and lateral view of flexion and extension, and by MRI and CT scans. The X-rays revealed multilevel (2–3 segments) degeneration with spondylolisthesis of a single segment. CT scans showed stenosis of the central vertebral canal and the lumbar lateral recess, but the degree of stenosis differed between individuals. It also demonstrated the degree of spondylolisthesis. MRI showed the extent of dural sac compression (Fig. 1). The levels responsible for the symptoms were determined from the radiological findings and a gait load test.

### Surgical technique

**Figure 2.**
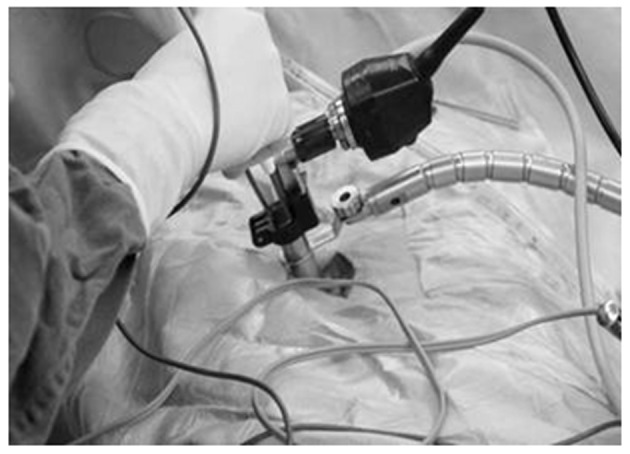
Microendoscopic discectomy (MED) was performed first for the segments with disc herniation.

**Figure 3.**
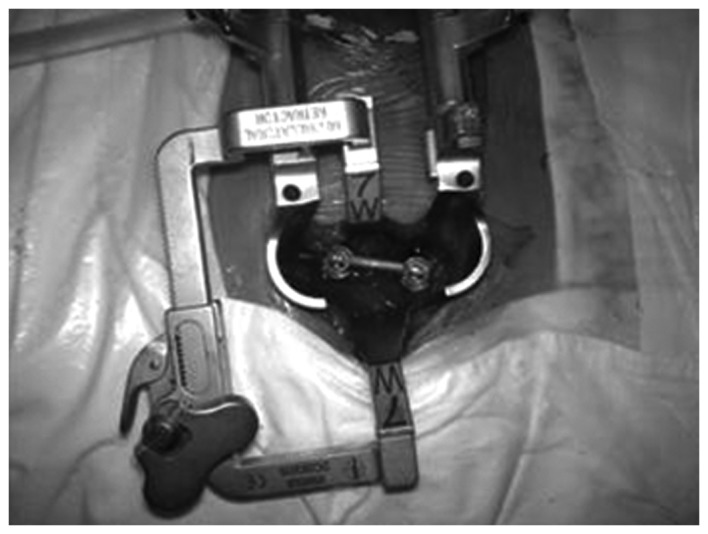
Minimally invasive transforaminal lumbar interbody fusion (MI-TLIF) was performed for the segment with spondylolisthesis by the paraspinal approach via a Quadrant working channel using a single cage and pedicle rod-screw system.

The surgery was performed under general anesthesia. The patients were placed in a prone position. In the traditional group, a posterior midline incision was made to access the lamina of the responsible segments, then laminectomy was performed to decompress the nerve roots. Posterior segmental spinal instrumentation was used in all cases, using a single cage and pedicle rod-screw system. In the minimally invasive group, a standard posterior midline incision was also made and a microendoscope was used first to decompress the stenosis and remove the herniated disc (Fig. 2). To treat the spondylolisthesis, a paraspinal approach was used to perform a MI-TLIF using a single cage and pedicle rod-screw system with the Quadrant system (an instrument with a working channel; Fig. 3).

### Postoperative care

The time of bed rest was 15–45 days in the traditional group and 5–7 days in the minimally invasive group. Routine antibiotics were administered for 1–2 days to prevent infection. Care was taken of the sensory functions and movement of the bilateral lower extremities and drainage of the incision. Back muscle exercise began when the incision pain had alleviated.

## Results

We evaluated the incision length, blood loss, time of bed rest and ODI score prior to and following surgery of the patients in the two groups. The data were statistically analyzed, using SPSS 13.0 statistical software. The measurement data were recorded, a t-test was performed to compare data from the two groups and P<0.05 was considered to indicate a statistically significant difference. In the minimally invasive group, the average length of the incision was 6.9±1.1 (6–11) cm, the surgery time was 232±28 (210–300) min, the blood loss was 361±122 (290–470) ml, the bed rest time was 6.1±0.8 (5–7) days and the ODI score was 40.3±6.7 preoperatively and 11.5±3.8 postoperatively. In the traditional group, the average length of the incision was 16.3±1.6 (16–22) cm, the surgery time was 204±21 (180–240) min, the blood loss was 610±194 (410–950) ml, the bed rest time was 23.7±9.9 (15–45) days, and the ODI scores were 39.4±7.2 and 13.6±3.1, respectively, prior to and following surgery. There were statistically significant differences in incision length, blood loss and bed rest time following surgery between the two groups (P<0.05). However, the surgery times and ODI scores of the two groups revealed no significant differences (P>0.05; Table I).

## Discussion

From the viewpoint of pathological anatomy, lumbar spinal stenoses are commonly multilevel, but rarely affect the whole lumbar spine. The junctional part of two segments, that is, the area between the upper segment and the lower one, including the articular process, intervertebral disc, ligamentum flavum and the connective parts of the upper and lower laminar, has the functions of movement and stability and is most vulnerable to degeneration (1). In the lumbar spinal canal, stenosis rarely occurs on the vertebral body and cross-section of the pedicle, but particularly affects the articular process, intervertebral disc and upper part of the laminar junction (2,3). According to anatomical pathology studies, the areas demanding decompression are localized on the canal near the intervertebral space, which provides the possibility of preserving the bony canal behind the vertebral body. The stability of the spine has been reported to be preserved due to the good preservation of the laminar and articular processes (2,3). These results provide theoretical evidence to support the limited decompression of the spinal canal.

DLSS is often characterized as multilevel from the radiologic findings, and has complicated and non-typical clinical manifestations, which make diagnosis and treatment difficult. According to a study by Park *et al*(4) of 13 spine surgery centers and 1091 patients from the USA, the patients who had spinal stenosis of more than 3 segments were most likely to be elderly males. Single-level stenosis is often located at the L4–L5 segment, while two-level stenosis typically affects L3–L5 and three-level stenosis affects L2–L5. Various surgical techniques for treating lumbar spinal stenosis have been reported. The standard surgery for the treatment of spinal stenosis at the early stage was extensive laminectomy for decompression. Currently, most spinal surgeons consider that extensive laminectomy destroys the stability of spine, and may lead to serious complications, including lumbar spondylolisthesis and epidural adhesions. Katz *et al*(5) confirmed that a quarter of the patients studied required further surgery and one-third complained of severe lower back pain during a 10-year follow-up. Thus, extensive laminectomy is no longer the standard treatment for spinal stenosis.

Since the main methods of the traditional PLIF technique are based on laminectomy or nerve root decompression to relieve the compression of the dural sac and nerve roots, the facet joint has often been removed with the lamina to ensure a full decompression, and damage to the stability of the spine is inevitable (5). The lack of protection for the lamina following decompression results in adhesion between the dural sac and nerve roots, which leads to more severe hyperostosis and re-stenosis of the spinal canal, and effects the long-term clinical outcomes. Denervation due to extensive stripping of the paraspinal muscle is also a major cause of prolonged lower back pain following surgery (6).

An increasing number of groups advocate complete decompression by limited, precise and more targeted techniques, rather than extensive resection, to maintain the stability of the spine by adopting less invasive techniques and to minimize the damage to the anatomical structure by avoiding preventive decompression at non-responsible levels (7–9). In terms of preserving the stability of the spine, the modified spinous-preserving laminectomy is preferable to total laminectomy, particularly since the bilateral decompression may be conducted using a unilateral approach, which has been reported in many studies and has achieved satisfactory clinical outcomes (10–12).

We recommend the following operative principles for the treatment of DLSS: locate the responsible level accurately, decompress the spinal canal effectively, damage the normal structure limitedly and avoid extensive resection. Limited decompression is achieved by the precise diagnosis and location of the responsible level according to the individual, and by preserving the stability and integrity of the posterior column of the spine so as to avoid the complications of iatrogenic instability and adhesion of dural scars. The key to improving the clinical outcomes is determining the responsible levels (13–15). The concept of precise spinal surgery was proposed at the 5th Chinese Orthopedic Association (COA) conference of the Chinese Medical Association by certain experts. The combined use of radiological examination and gait load testing for the clinical and functional examinations is likely to aid the clarification of the response level and range of positions that require decompression, so as to avoid the complication of iatrogenic instability. There is no need to carry out preventive surgery for radiologically diagnosed stenosis without clinical symptoms (13–15).

**Figure 4.**
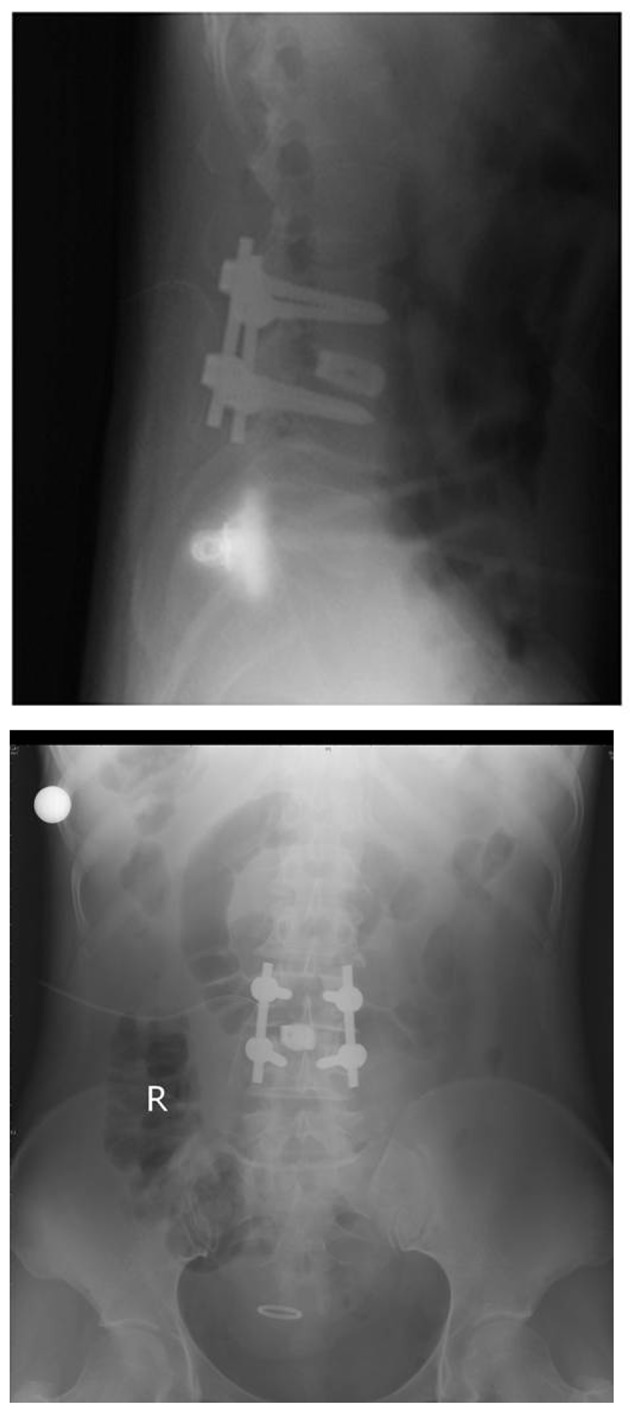
X-ray of the (A) lateral view and (B) AP view following minimally invasive transforaminal lumbar interbody fusion (MI-TLIF) surgery.

**Figure 5.**
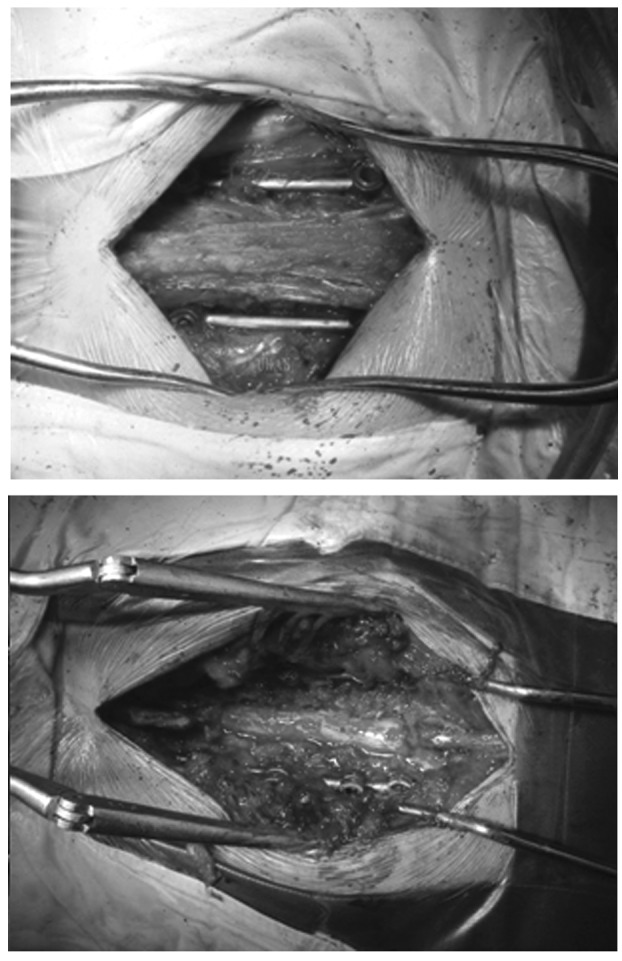
(A) The posterior column of spine was intact following surgery from a paraspinal approach using a pedicle screw system and (B) the posterior column of the spine was destroyed following traditional laminectomy.

MED from the posterior approach is a typical and internationally recognized minimally invasive technique for the treatment of lumbar disc herniation which has become increasingly popular and developed in recent years and may also be used for the minimally invasive laminectomy of lumbar spinal stenosis. It is characterized by reduced invasion, quicker recovery and improved clinical outcomes with clear advantages (16–18), but may not be used for the treatment of patients with spondylolisthesis. Compared with the traditional PLIF technique, the TLIF technique causes less destruction of the posterior column of the spine (19). In particular, the application of MI-TLIF for the treatment of lumbar spondylolisthesis by the paraspinal approach has the advantages of reduced blood loss and less damage to the paraspinal soft tissue (Figs. 4 and 5), but it also has the limitations of long surgery times and a complicated surgical technique (20–22). We have attempted to carry out a combination of MED and MI-TLIF techniques for the treatment of DLSS with spondylolisthesis. MED was used to decompress the nerve roots of the responsible level while MI-TLIF was performed on the segments with spondylolisthesis. Minimally invasive decompression and fusion may be carried out with a combination of these two minimally invasive techniques, so that the destruction of the posterior column of the spine is minimized. All 26 patients in the minimally invasive group reached satisfactory clinical improvements following surgery while also having reduced blood loss, less damage to the paraspinal soft tissue, improved outcomes and shorter recovery times than the patients treated with the PLIF technique and similar short-term clinical outcomes (Table I). The combination of MED and MI-TLIF is a minimally invasive technique for spinal surgery with clear advantages and bright prospects worthy of further study and promotion.

## Figures and Tables

**Table I.** Comparison between the minimal invasive (MED+TLIF) group and the traditional PLIF group.

Group (n)	Incision (cm)	Surgery time (min)	Blood loss (ml)	Bed rest time (days)	Pre-surgery ODI	Post-surgery ODI
MI-TLIF (26)	6.9±1.1^a^	232±28	361±122^a^	6.1±0.8^a^	40.3±6.7	11.5±3.8
PLIF (27)	16.3±1.6	204±21	610±194	23.7±9.9	39.4±7.2	13.6±3.1

MED, microendoscopic discectomy; TLIF, transforaminal lumbar interbody fusion; ODI, Oswestry Disability Index; PLIF, posterior lumbar interbody fusion; MI, minimally invasive.

a P<0.05.
